# Modelling Impact of Different Varicella Immunisation Strategies Upon Introduction in the Swedish National Programme

**DOI:** 10.1111/apa.70487

**Published:** 2026-03-25

**Authors:** Gianpaolo Scalia Tomba, Frida Kasteng, Annika Ersson, Sören Andersson, Emma Appelqvist, Marie Nordahl, Kari Johansen

**Affiliations:** ^1^ Department of Mathematics University of Rome Tor Vergata Rome Italy; ^2^ Department of Mathematics Stockholm University Stockholm Sweden; ^3^ Department of Public Health Analysis and Data Management Public Health Agency of Sweden Solna Sweden; ^4^ School of Medical Sciences, Faculty of Medicine and Health Örebro University Örebro Sweden; ^5^ Department of Global Public Health Karolinska Institutet Stockholm Sweden

**Keywords:** burden of disease, catch‐up vaccination, immunisation strategies, impact, modelling, varicella vaccine

## Abstract

**Aim:**

Countries with varicella immunisation programmes have seen major reductions in disease burden. While two‐dose schedules are now universally adopted, dosing ages vary, and some countries initially include catch‐up vaccination of older children to speed impact. This modelling study assessed three two‐dose schedules in the Swedish setting (1) 12 and 18 months, (2) 18 months and 5 years, and (3) 18 months and 7 years, with and without catch‐up of susceptible children based on their medical history.

**Methods:**

A deterministic, compartmental, age‐structured, dynamic transmission model was used to evaluate the different strategies.

**Results:**

With coverage similar to other childhood vaccinations in Sweden (≥ 95%) but no catch‐up, varicella cases are projected to decrease significantly within four years of programme start for all schedules modelled. However, a temporary rebound may occur around years 4–7 due to accumulating susceptibles before subsiding. Adding catch‐up vaccination in this high‐coverage setting accelerates and sustains the decline, with varicella disease burden significantly reduced within 2–3 years.

**Conclusion:**

A national childhood varicella programme in Sweden could cut transmission and disease burden within 4 years, with catch‐up vaccination speeding this to a few years and lowering rebound risk. The schedule should be guided by programmatic considerations.

## Summary

- This dynamic transmission modelling study showed that a two‐dose varicella vaccination schedule in Sweden could markedly reduce disease burden within 4 years across multiple timing options, assuming high coverage.
- Early catch‐up vaccination would speed impact to 2–3 years and help prevent rebound outbreaks.
- Schedule choice should reflect programmatic feasibility, visit burden, parental preferences, prior private vaccination, and the epidemiological need for early protection, including before international travel.

**Abbreviations**
EU: European Union
HZ: herpes zoster
MMR: measles, mumps, and rubella‐containing combination vaccine
MMRV: measles, mumps, rubella, and varicella‐containing combination vaccine
V: monovalent varicella‐containing vaccine
VZV: varicella zoster virus

## 1 Introduction

In 1974, a live attenuated varicella vaccine (containing the Oka strain) was developed for the prevention of varicella, initially targeting high‐risk children [1]. The vaccine later became commercially available, and in 1987, a single‐dose schedule for children over the age of one year who had no history of varicella infection was recommended in Japan [2, 3].

Subsequently, the number of countries that introduced varicella vaccines into national immunisation programmes continued to increase over the following decades, and in 2024, 47 countries recommend varicella vaccination according to the World Health Organization, with more countries currently assessing an introduction [4]. Following reports of breakthrough infections in early adopter countries such as in the United States and Germany, a second dose was introduced in 2006 and 2009, respectively [5, 6]. While two‐dose schedules are now universally adopted, the recommended ages for the first and second doses vary by country [7]. The first dose is typically administered between 11 and 18 months of age, and the second dose between 15 months and 7 years of age. Varicella vaccination has often, but not always, followed the age for administration of the MMR vaccine.

Additionally, some countries have implemented or plan for implementing catch‐up vaccination of older susceptible children due to no previous infection or vaccination, alongside the initiation of national immunisation programmes avoiding the risk of postponing the varicella infection to older age groups and leading to a more rapid reduction in the burden of varicella disease [8, 9]. An early modelling study from 1996 suggested the importance of adding catch‐up vaccination to older susceptible individuals to avoid increasing the age of varicella cases if routine programmes mainly target pre‐school children [10]; however, few countries followed this approach upon introduction of varicella vaccination. A later modelling study from Finland, published in 2010, suggested that incorporating catch‐up vaccination would more rapidly reduce the number of varicella cases compared to routine vaccination alone [11]. These modelling results were later confirmed following the introduction of Finland's national varicella immunisation programme in 2017, which included a 6‐month catch‐up vaccination period targeting older children up to 11 years of age [9]. Similarly, the Navarra region in Spain carried out catch‐up vaccination for children up to 10 years of age during the initial phase of its national varicella immunisation programme, resulting in a rapid decline in varicella cases [12].

The original Oka vaccine virus strain is today included in all four varicella‐virus‐containing vaccines available within the EU, and more than 30 million individuals globally have received the Oka strain‐containing vaccines, either as a monovalent varicella vaccine (V) or as part of a tetravalent combination vaccine with measles, mumps, rubella, and varicella viruses (MMRV) [3].

In many national immunisation programmes, the first dose is administered as a monovalent varicella vaccine, either alone or simultaneously with the MMR vaccine (V or V + MMR) [13]. Occasionally, the tetravalent vaccine (MMRV) is used for the first dose. For the second dose, the MMRV combination vaccine is more commonly used, if available [13]. However, in some countries, supply shortages have led to the continued use of the monovalent vaccine alone (V) or in combination with the MMR vaccine (V + MMR) for both doses [13].

Following assessments of varicella vaccine safety, efficacy, effectiveness, and impact in randomised clinical trials and observational studies [14], as well as logistical and programmatic aspects regarding the Swedish immunisation schedule for children, a modelling study was conducted to evaluate the impact on burden of varicella disease using different vaccination strategies in the Swedish context. Further, since more evidence to examine how different varicella vaccine coverage levels would change varicella epidemiology was requested in the 2014 Background Paper on Varicella Vaccine by the WHO SAGE Working Group on Varicella and Herpes Zoster, this was also evaluated [15].

The schedules examined included administering the two doses at: (1) 12 and 18 months of age, (2) 18 months and 5 years of age, and (3) 18 months and 7 years of age. Earlier, a modelling study has assessed the impact in Sweden using only the schedule 12 and 18 months of age [16] but this time further schedules were also considered to explore options to vaccinate at already scheduled visits also in older children. Earlier serological data from the Swedish setting and other Nordic settings suggest that among older children and adolescents from 12 years of age approximately 10% have no antibodies to varicella and therefore are expected to have no protection against disease [17, 18]. In addition, it is new that among the recent increase in number of newcomers into the country from geographical settings where varicella spreads first more often in older age groups, susceptibles often can be found among children, adolescents, and adults [18]. Each schedule assessed was therefore analysed both with and without the inclusion of catch‐up vaccination up to 18 years of age.

## 2 Methods

### 2.1 Epidemiological Modelling

The main purpose of the modelling study was to evaluate differences in the impact on burden of varicella disease between three plausible varicella vaccination schedules targeting different age groups in the Swedish setting (1) 12 and 18 months, (2) 18 months and 5 years, and (3) 18 months and 7 years, with and without catch‐up of susceptible children based on their medical history see Table 1. A deterministic, compartmental, age‐structured, dynamic transmission model was used [16]. A systematic review of the literature was conducted jointly with public health agencies in the Nordic countries prior to this work (Prospero ID CRD42023416345), focusing on the methods in previous modelling studies as well as the assumptions made in these studies and any changes in the evidence base behind them.

**TABLE 1:** Impact on disease burden was modelled using three immunisation schedules in routine schedules with no or with catch‐up vaccination.

Modelled scenarios	Varicella vaccination in routine schedule WITH NO catch‐up vaccination of older naïve children/adolescents (age at vaccination)	Varicella vaccination in routine schedule WITH catch‐up vaccination of older naïve children/adolescents^a^ (age at vaccination)
1.	Dose 1: 12 months Dose 2:18 months	Dose 1: 12 months Dose 2: 18 months Catch‐up: 5 + 7 years, 7 + 11 years, or 11 + 13 years
2.	Dose 1: 18 months Dose 2: 5 years	Dose 1: 18 months Dose 2: 5 years Catch‐up: 5 + 7 years, 7 + 11 years, or 11 + 13 years
3.	Dose 1: 18 months Dose 2: 7 years	Dose 1: 18 months Dose 2: 7 years Catch‐up: 5 + 7 years, 7 + 11 years, or 11 + 13 years

^a^ In the catch‐up programme vaccination, two doses of varicella vaccine may be offered also to older susceptible children during planned healthcare visits without vaccinations at e.g., 13 + 15 years of age. For older adolescents, it needs to be planned individually when need arises in the age group 15–18 years with at least one month between dose 1 and 2.

To include all relevant effects of the vaccination, the time horizon was set to 95 years. This allowed the model to account for the long‐term impact of vaccination regarding the health effects that might arise also decades after a VZV infection as the vaccine virus remains latent in the body and may cause HZ later in life.

Protection from maternal antibodies was assumed until three months of age, after which susceptibility to VZV was expected [19]. Force of infection and contact rate were calibrated to fit Swedish seroprevalence data for children up to age 12, matched with Finnish data for older age groups [20]. Contact patterns were based on a synthetic matrix that described the intensity of total contacts between age groups.

An overview of the epidemiological transmission model used is shown in Figure 1.

**FIGURE 1:**
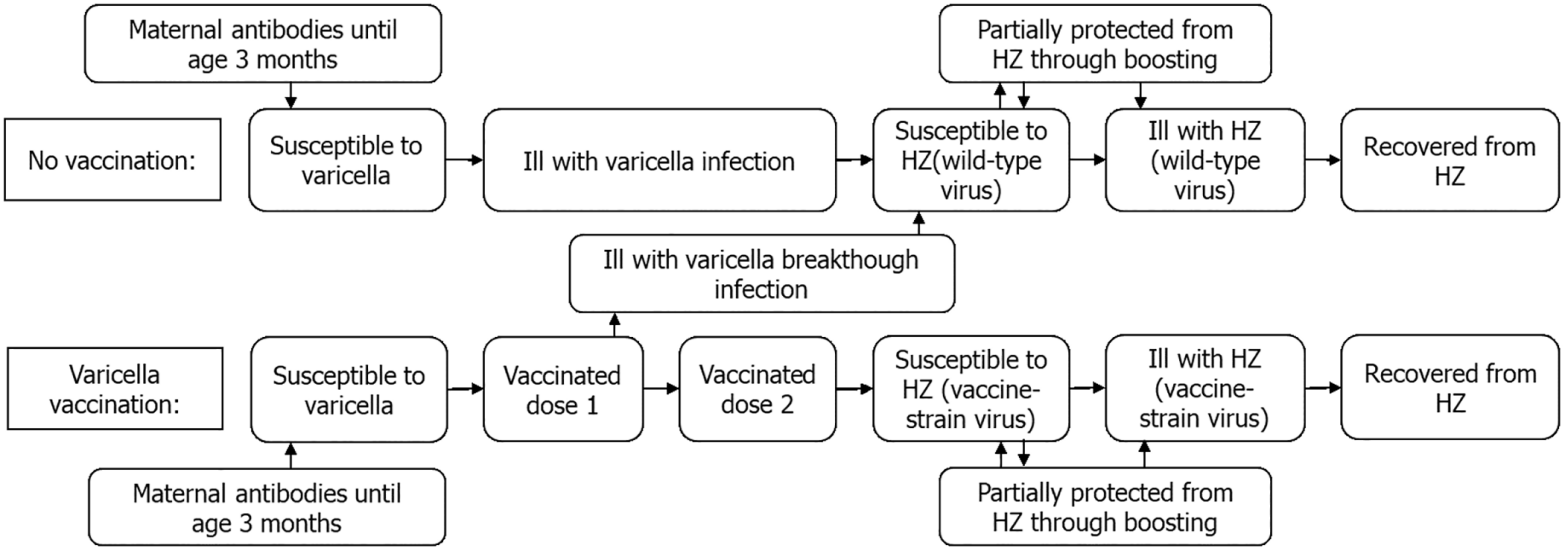
Flowchart of the epidemiological model used in a deterministic, compartmental, age‐structured, dynamic transmission model.

### 2.2 Parameters and Assumptions

#### 2.2.1 Incidence of Varicella and HZ

A constant birth rate of approximately 120 000 children per year was assumed.

Varicella incidence in different age groups was based on a study of 957 blood samples collected from children aged 0–5 years and 12–13 years during the time period 2011–2013, being the most recent material at the time of this modelling study available for Swedish children [18]. The seroprevalence had reached 67% in 5‐year‐olds and 92% in 12‐year‐olds using the ELISA Enzygnost Anti‐VZV/IgG assay. An earlier study assessing seroprevalence with an in‐house ELISA over time from the 1960s and onward was mostly in line with the latest and showed an increase of seropositivity over the years for VZV for 9–12 year old children; 50% in 1967–1968, 74%–82% in 1977–1978, and 98% in 1997 [17]. For the older children, data from Finland, collected in 1997–1998, were used in the current modelling to estimate seroprevalence in older age groups [20]. In this Finnish dataset, 93% of 10–14‐year‐olds and 97% of those aged 15–29 years were seropositive.

It is possible that immigration has affected the seroprevalence in the Swedish population in later years after 2013; however, the number of hospitalisations has remained much the same over the last 20 years and could therefore serve as a proxy indicating that these seroprevalence data are still valid.

We included HZ incidence in the model due to the impact of varicella vaccination on future HZ susceptibility in vaccinated individuals and the possible effect of exogenous boosting from VZV on HZ susceptibility. The number of primary care visits due to HZ was used as a proxy for HZ incidence from a study using primary care data for the period 2008–2010, from the Region Västra Götaland, one of the larger regions in Sweden population‐wise, with nearly one‐fifth of the national population [21]. We assumed the same age‐adjusted incidence rates at national level.

### 2.3 Impact of Intervention—Vaccine Effectiveness

The effectiveness of varicella vaccination was set at the estimates according to available post‐licensure follow‐up data in our own group and reported on vaccine effectiveness 81% (95% CI: 78–84) for the first dose with a 2% annual waning rate, and at 92% (95% CI: 88–94) for the second dose with no waning effect [14]. We assumed the same effectiveness against varicella for monovalent and tetravalent vaccines. Varicella infection following vaccination was referred to as a breakthrough infection and assumed to be mild, i.e., not incurring a risk of specialised or inpatient care in the model. We modelled the risk of HZ in vaccinated individuals over the 95‐year time horizon of the model. The HZ reactivation risk with the vaccine strain virus was set at 10% of the risk with wild‐type viruses. This figure was derived from follow‐up data in varicella‐vaccinated children up to the age of 18 years in the USA [22].

### 2.4 Vaccination Coverage

Vaccination coverage was assumed to be 95% for both the national vaccination programme and the catch‐up vaccination strategy, based on the vaccination coverage for the routine MMR vaccination programme over time in the same age groups in Sweden [23]. This assumption was varied in sensitivity analyses at 95%, 85%, and 75% coverage in the most likely vaccination strategy scenario with catch‐up following a first modelling exercise where vaccination coverage in the lower end at 80%, 70%, and 60% was assessed. The same coverage was assumed for doses 1 and 2.

### 2.5 Population Immunity

The model accounted for population immunity effects of vaccination since the force of infection is proportional to both the number of infected and susceptible individuals. Population immunity occurs when there are too few susceptible (because of high coverage of vaccination) to sustain disease transmission. It should be noted that social structure, which is not explicitly represented in the model, is important for the population immunity effect. Thus, even if population immunity is generally applicable, small closed susceptible communities may still experience outbreaks.

### 2.6 Exogenous Boosting

Exogenous boosting refers to protection against HZ due to immune system stimulation after re‐exposure to VZV in previously infected individuals [24]. The relevance of exogenous boosting has been discussed, and no increase in the number of herpes zoster cases has been observed in the countries that included varicella vaccination now 20–30 years ago. In fact, among the newly vaccinated individuals, a reduction in herpes zoster has been documented in registry studies up to age 18 years [22, 25, 26]. More long‐term studies are needed and expected to be conducted in the countries that introduced vaccination early on and have access to large health care databases available for registry studies. However, in the base case analysis of this study, exogenous boosting is assumed to fully prevent HZ during 4 years following exposure to VZV among seropositive individuals [16].

## 3 Results

In the transmission model, we first analysed implementing a two‐dose varicella vaccination programme, with the first dose administered either at 12 or 18 months and the second dose either at 18 months, 5 years, or 7 years. Varicella disease is projected to decrease significantly, by approximately 85%, within 4 years of programme initiation, irrespective of which of the tested three schedules is chosen. See Figure 2a.

**FIGURE 2:**
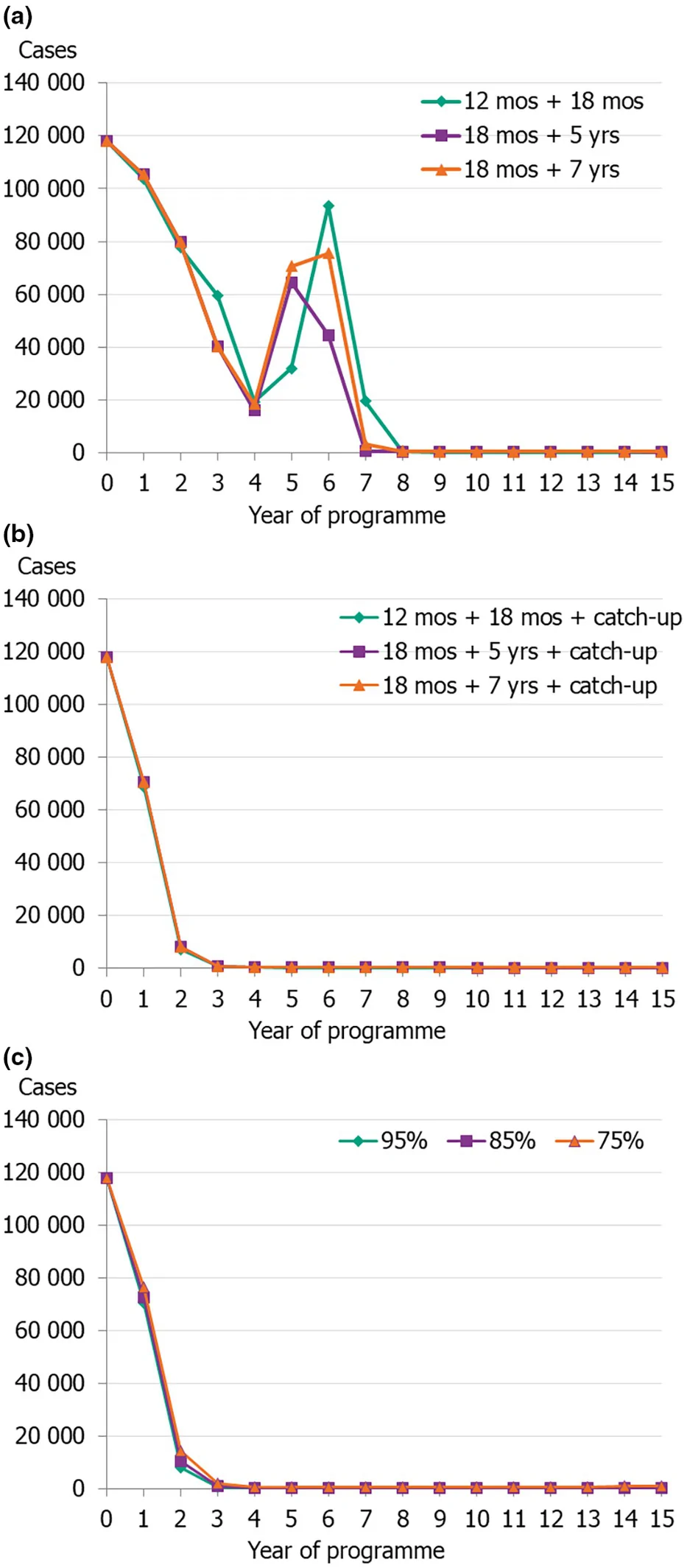
(a) Estimated number of varicella cases in all age groups following introduction of varicella vaccination in the three chosen immunisation schedules; (a) 12 and 18 months of age, (b) 18 months and 5 years of age, and (c) 18 months and 7 years of age. (b) Estimated number of varicella cases in all age groups following introduction of varicella vaccination in the three chosen immunisation schedules; (a) 12 and 18 months of age, (b) 18 months and 5 years of age, and (c) 18 months and 7 years of age with catch‐up vaccination of older susceptible children. (c) In a sensitivity analysis, the impact of lower vaccination coverage on the estimated number of varicella cases in all age groups has been analysed for one of the immunisation schedules 18 months and 7 years with catch‐up of older susceptible children following the introduction of varicella vaccination. A lower vaccination coverage of 85, or even 75%, would also reduce the number of cases to less than 1000 cases per year after 3 years; however, the model simulates an even uptake across the population and does not take into account import cases.

However, a *rebound* period with a higher number of cases is expected to be seen between years 4 and 7, as the number of susceptible individuals increases, before the disease eventually subsides. In the end, the number of varicella cases will be reduced from over 100 000 per year, to only a few hundred seen yearly. See Figure 2a.

In contrast, implementing also catch‐up vaccination for older children during the initial years of the routine programme leads to a more rapid and sustained reduction in varicella cases, with no rebound period and a disease burden of less than 1000 cases per year expected within 2–3 years, irrespective of which immunisation schedule is chosen. See Figure 2b.

In a sensitivity analysis, the impact of lower vaccination coverage was analysed for one of the immunisation schedules: 18 months and 7 years with catch‐up of older susceptible children, which may be the most likely programme in Sweden since the two doses of MMR vaccine are offered at these two ages and therefore may be the chosen schedule when varicella vaccination is introduced. See Figure 2c. A lower vaccination coverage of 85, or even 75%, would also reduce the number of cases to less than 1000 cases per year after 3 years; however, the model simulates an even uptake across the population; thus, lower coverage rates may be associated with a higher risk of outbreaks in population groups with suboptimal coverage and does not take into account import cases. The minor differences that can be perceived at years 1 and 2 for the different vaccination schedules with catch‐up vaccination are as expected: lower coverage yielding slightly more cases, but the difference is just a few thousand cases and should be considered not significant given the model uncertainty. However, in the initial modelling exercise, 70% and 60% vaccination coverage, especially if the vaccines were offered at the more convenient time points: 18 months and 7 years together with MMR, significantly increased the risk of epidemics with both natural infections and breakthrough infections. See Figure 1a–c in Supplement.

## 4 Discussion

Varicella vaccination has significantly reduced disease burden in countries with national immunisation programmes; however, an evaluation has to be performed in each country considering an introduction of varicella immunisation. This modelling study evaluated in a Swedish setting various common vaccination schedules, in combination with or without catch‐up vaccination of older susceptible children. The results, consistent with other modelling studies and real‐world data, show that routine childhood vaccination can nearly eliminate varicella within a short time frame, provided vaccine coverage is high.

Varicella is highly contagious, with an estimated R_0_ of 3.3–16.9 assessed across 11 European countries within the ESEN‐2 project [20]. Sweden did not participate in this study, but R_0_ was estimated to be 4.85 (95% CI 3.89–6.04) in the neighbouring country Finland [20]. In Sweden, the median age of infection is around 4 years [27]. While the disease often is mild in children and does not require medical care, the high incidence of varicella infections leads to a notable burden, including the number of complications like secondary bacterial infections, septicemia, pneumonia, and hospitalisations (the latter approximately 0.2% of all cases in Sweden). In addition, encephalitis may occur rarely and cause long‐term sequelae, and varicella has also been linked to an increased risk of stroke in children, especially in the first year post‐infection.

Varicella vaccines, available as monovalent or in combination with other vaccines, are safe and effective. They reduce not only the number of infections but also the risk of complications including development of herpes zoster. Countries that have introduced varicella vaccination into their routine immunisation schedules have taken different approaches regarding timing and intervals between doses. Some use a short interval (e.g., two doses before age 2), while others use a longer interval, with the second dose administered up to 6 years after the first. Whether the interval between doses matters for long‐term immunity and protection against varicella and herpes zoster during childhood or later is still unknown, but there is evidence that two doses are needed to avoid breakthrough infections.

Modelling shows that if a routine 2‐dose schedule is used, varicella virus circulation will decrease over time; however, subsequently the pool of susceptible older children is large enough to support a rebound in varicella cases several years after a vaccination programme is initiated. However, this risk can be mitigated by introducing catch‐up vaccinations for susceptible older children, thereby reducing community circulation more rapidly.

Most studies estimate that coverage of 80%–90% is needed to eliminate virus circulation in the community, though higher levels are always preferable [7]. Population immunity thresholds were analysed in the ESEN2‐study and ranged from 69.8 (95% CI 64.5–73.9) in Italy and were highest in the Netherlands 94.1 (95% CI 91.3–95.9), the only country above 90%. Our close neighbour Finland, with rather similar patterns of social contacts, estimated a threshold of 79.4 (95% CI 74.3–83.4).

Offering catch‐up vaccination to older susceptible children alongside routine childhood vaccination accelerates the reduction in disease burden, regardless of the schedule used. It also prevents the risk of case rebounds. This was demonstrated in real life upon introduction of varicella vaccination and reported from the Navarra region in Spain (2013) and in Finland (2017) at the time of introduction [8, 9]. Recent UK modelling has reached similar conclusions [28], and catch‐up vaccination is planned alongside the upcoming routine introduction [29].

In our model, catch‐up vaccination was aligned with existing scheduled visits at ages 5, 7, 11, or 13, with the second dose given at later routine visits (e.g., 7, 9, 11, or 15 years). For those over 13, individual planning is needed, though school health services can support delivery. This low‐intensity, gradual approach requires several years. A faster rollout, like Finland's 6‐month implementation, could reduce disease more quickly but would be more resource‐intensive, though offering earlier protection and a shorter implementation period. Offering the catch‐up vaccine via child and school health services is expected to result in a more equal vaccine coverage compared to an organisation where care‐givers' understanding and initiative are of higher importance in order for the child to be vaccinated.

If catch‐up vaccinations are not integrated into the existing routine vaccination programme and the full effect of the routine programme will be delayed, there is a risk of varicella infections in older susceptible children, adolescents, and later young adults. In this situation, many parents may opt for private vaccination for the first, the second, or both doses, resulting in unequal coverage most likely due to socioeconomic reasons and, in the end, may hinder achieving the aim of high protection levels.

In addition, varicella tends to be more severe in adolescents and adults, with hospitalisation rates twice as high as in children under 15 [30]. Also, pregnant women are especially vulnerable: infection during the first 20 weeks can affect fetal development [31] and infection around delivery poses serious risks. Maternal complications may include pneumonia or encephalitis, while newborns risk congenital varicella syndrome or severe neonatal infection.

Historically, Sweden has maintained high vaccination coverage through the well‐functioning child health and school‐based programmes, with approximately 95% coverage for vaccines like MMR doses 1 and 2, and even higher for the other paediatric immunisation programme vaccines [23, 32]. Caregivers generally express confidence in the vaccination programme, supporting the high vaccination coverage achieved in Sweden [33]. Research on vaccine acceptance of Swedish parents regarding varicella vaccination is limited. A 2018 study (not previously published) of 1056 parents participating in a web‐panel run by the Public Health Agency of Sweden (75% response rate) found that most parents were positive (46%) or neutral (39%) to have their child vaccinated against varicella. Of the parents, 15% had a negative response to having their child vaccinated. If the vaccine were to be included in the national programme for children under the age of 2 years, 86% of parents with children aged 0–5 responded that they intended to get their child vaccinated. Among parents of older children (6–15 years), 79%–87% were positive to vaccinating younger children as part of the national programme. Almost all parents, 96%, were positive to have their child vaccinated as teenagers as part of a national programme, if their child had not had any prior varicella infection.

A recent UK study found 74% of parents were likely to accept varicella vaccination, 18% were unlikely, and 8% were undecided. A systematic review identified common concerns, including the belief that varicella is mild, doubts about vaccine efficacy and safety, preference for natural immunity, and the strong role of parental decision‐making influenced by social factors. These findings underscore the need to understand country‐specific facilitators and barriers to both routine and catch‐up vaccination.

Finally, documenting varicella vaccine doses in national immunisation registries is essential for both routine and catch‐up programmes to support long‐term stakeholder coordination and track the impact on herpes zoster. In many countries, adult vaccination data are often missing. In Sweden, comprehensive documentation would require government action and changes to the Infectious Disease Act.

In conclusion, high‐coverage routine childhood varicella vaccination, ideally combined with catch‐up programmes over a shorter or longer time period, can significantly reduce the burden of varicella disease. Ensuring national documentation of varicella vaccination across all age groups, as well as public understanding and support for the programme, will be key to achieving successful implementation.

## 5 Limitations

Study limitations include the inherent simplifications of modelling, though similar results across countries and methods support the findings. The analysis focused on vaccination schedules deemed feasible within the Swedish context, based on the current immunisation programme and existing data. The impact of private vaccinations, including those before travel, import of varicella cases, and migration of susceptible individuals over time, was not considered.

## 6 Conclusion

This modelling study shows that adding catch‐up vaccination in combination with any of the analysed immunisation schedules can reduce varicella disease within 2–3 years, compared to 4–7 years without it. The choice of the schedule should be guided by programmatic factors, including minimising extra visits, caregiver preferences, and protection during travel.

## Author Contributions

**Gianpaolo Scalia Tomba:** conceptualization, methodology, investigation, formal analysis, writing – review and editing. **Frida Kasteng:** methodology, investigation, formal analysis, writing – review and editing. **Annika Ersson:** writing – review and editing. **Sören Andersson:** conceptualization, writing – review and editing. **Emma Appelqvist:** methodology, investigation acceptance study in support of expected vaccine coverage. **Marie Nordahl:** writing – review and editing. **Kari Johansen:** conceptualization, writing – original draft, writing – review and editing.

## Funding

The study was supported entirely by the Public Health Agency of Sweden, Solna, Sweden.

## Conflicts of Interest

The authors declare no conflicts of interest.

## Supporting information


**Appendix S1:** Supporting Information.

## Data Availability

The data utilized comes from requests by the Public Health Agency of Sweden to the National Board of Health and Welfare.
