# Supplementary material for: Modelling Impact of Different Varicella Immunisation Strategies Upon Introduction in the Swedish National Programme
Source: Acta Paediatr. 2026 Mar 25;115(11):2525–32. doi: 10.1111/apa.70487 (PMC13659423; doi:10.1111/apa.70487)
Supplement: Supplementary file 1 [file APA-115-2525-s001.docx]

SUPPLEMENT TO MAIN ARTICLE

In figure 1a-1c the impact of varicella vacccinaiton on reduction of number of cases of varicella at different vaccination coverage scenarios 80, 70 and 60%, when no catch-up vaccination of older susceptible children is made available and shows that at least 80% vaccination coverage should be recommended.

Supplemental Figure 1a

80%

|  | 5(80%) | 2(80%) |
| --- | --- | --- |
| 0 | 117980 | 117980 |
| 0 | 117980 | 117980 |
| 0 | 117980 | 117980 |
| 0 | 117980 | 117980 |
| 0 | 117980 | 117980 |
| 1 | 107076 | 106019 |
| 2 | 84550 | 80894 |
| 3 | 48075 | 62276 |
| 4 | 23391 | 21341 |
| 5 | 62359 | 30740 |
| 6 | 88029 | 97186 |
| 7 | 5473 | 22042 |
| 8 | 847 | 806 |
| 9 | 794 | 466 |
| 10 | 821 | 433 |
| 11 | 847 | 438 |
| 12 | 871 | 441 |
| 13 | 876 | 447 |
| 14 | 849 | 441 |
| 15 | 823 | 426 |
| 16 | 816 | 420 |
| 17 | 815 | 421 |
| 18 | 820 | 423 |
| 19 | 830 | 429 |
| 20 | 838 | 439 |
| 21 | 846 | 448 |
| 22 | 853 | 457 |
| 23 | 860 | 467 |
| 24 | 869 | 476 |
| 25 | 876 | 486 |
| 26 | 885 | 496 |
| 27 | 893 | 506 |
| 28 | 901 | 516 |
| 29 | 912 | 527 |
| 30 | 921 | 538 |
| 31 | 929 | 548 |
| 32 | 938 | 559 |
| 33 | 944 | 570 |
| 34 | 951 | 579 |
| 35 | 957 | 589 |
| 36 | 962 | 599 |
| 37 | 966 | 607 |
| 38 | 973 | 616 |
| 39 | 977 | 626 |
| 40 | 979 | 634 |
| 41 | 980 | 640 |
| 42 | 978 | 646 |
| 43 | 973 | 650 |
| 44 | 967 | 651 |
| 45 | 964 | 654 |
| 46 | 964 | 657 |
| 47 | 962 | 659 |
| 48 | 958 | 660 |
| 49 | 953 | 660 |
| 50 | 948 | 661 |
| 51 | 940 | 660 |
| 52 | 931 | 657 |
| 53 | 922 | 652 |
| 54 | 911 | 647 |
| 55 | 899 | 641 |
| 56 | 884 | 634 |
| 57 | 868 | 625 |
| 58 | 846 | 611 |
| 59 | 822 | 596 |
| 60 | 796 | 578 |
| 61 | 765 | 557 |
| 62 | 732 | 533 |
| 63 | 698 | 508 |
| 64 | 665 | 482 |
| 65 | 634 | 459 |
| 66 | 604 | 436 |
| 67 | 575 | 414 |
| 68 | 545 | 392 |
| 69 | 516 | 371 |
| 70 | 488 | 350 |
| 71 | 461 | 330 |
| 72 | 435 | 312 |
| 73 | 409 | 292 |
| 74 | 383 | 274 |
| 75 | 359 | 256 |
| 76 | 333 | 238 |
| 77 | 309 | 220 |
| 78 | 284 | 201 |
| 79 | 259 | 184 |
| 80 | 235 | 165 |
| 81 | 211 | 150 |
| 82 | 189 | 133 |
| 83 | 166 | 116 |
| 84 | 146 | 100 |
| 85 | 125 | 85 |
| 86 | 106 | 72 |
| 87 | 88 | 59 |
| 88 | 73 | 47 |
| 89 | 61 | 38 |
| 90 | 50 | 30 |
| 91 | 40 | 23 |
| 92 | 32 | 18 |
| 93 | 26 | 14 |
| 94 | 21 | 10 |
| 95 | 17 | 8 |

Supplemental Figure 1b

70%

|  | 5(70%) | 2(70%) |
| --- | --- | --- |
| 0 | 117980 | 117980 |
| 0 | 117980 | 117980 |
| 0 | 117980 | 117980 |
| 0 | 117980 | 117980 |
| 0 | 117980 | 117980 |
| 1 | 108321 | 107233 |
| 2 | 87982 | 83183 |
| 3 | 54137 | 64477 |
| 4 | 28547 | 23434 |
| 5 | 58580 | 29498 |
| 6 | 96255 | 98757 |
| 7 | 9629 | 24588 |
| 8 | 1467 | 1132 |
| 9 | 1379 | 628 |
| 10 | 1646 | 578 |
| 11 | 2037 | 632 |
| 12 | 2551 | 685 |
| 13 | 3169 | 751 |
| 14 | 3547 | 807 |
| 15 | 3576 | 807 |
| 16 | 3623 | 782 |
| 17 | 3776 | 835 |
| 18 | 3960 | 884 |
| 19 | 4153 | 929 |
| 20 | 4324 | 971 |
| 21 | 4466 | 1011 |
| 22 | 4576 | 1045 |
| 23 | 4644 | 1072 |
| 24 | 4695 | 1094 |
| 25 | 4746 | 1117 |
| 26 | 4805 | 1139 |
| 27 | 4873 | 1161 |
| 28 | 4955 | 1185 |
| 29 | 5060 | 1211 |
| 30 | 5174 | 1239 |
| 31 | 5290 | 1266 |
| 32 | 5412 | 1294 |
| 33 | 5534 | 1324 |
| 34 | 5652 | 1352 |
| 35 | 5775 | 1381 |
| 36 | 5905 | 1411 |
| 37 | 6044 | 1440 |
| 38 | 6196 | 1468 |
| 39 | 6342 | 1500 |
| 40 | 6478 | 1527 |
| 41 | 6611 | 1554 |
| 42 | 6738 | 1579 |
| 43 | 6841 | 1601 |
| 44 | 6908 | 1615 |
| 45 | 6976 | 1631 |
| 46 | 7067 | 1647 |
| 47 | 7166 | 1661 |
| 48 | 7256 | 1673 |
| 49 | 7321 | 1682 |
| 50 | 7373 | 1689 |
| 51 | 7418 | 1695 |
| 52 | 7454 | 1697 |
| 53 | 7485 | 1697 |
| 54 | 7507 | 1695 |
| 55 | 7524 | 1687 |
| 56 | 7544 | 1679 |
| 57 | 7564 | 1667 |
| 58 | 7572 | 1648 |
| 59 | 7566 | 1621 |
| 60 | 7545 | 1587 |
| 61 | 7510 | 1547 |
| 62 | 7462 | 1497 |
| 63 | 7403 | 1442 |
| 64 | 7338 | 1383 |
| 65 | 7272 | 1326 |
| 66 | 7210 | 1270 |
| 67 | 7151 | 1215 |
| 68 | 7088 | 1160 |
| 69 | 7019 | 1103 |
| 70 | 6948 | 1047 |
| 71 | 6873 | 994 |
| 72 | 6796 | 941 |
| 73 | 6720 | 889 |
| 74 | 6644 | 837 |
| 75 | 6569 | 786 |
| 76 | 6494 | 736 |
| 77 | 6418 | 685 |
| 78 | 6342 | 633 |
| 79 | 6265 | 581 |
| 80 | 6188 | 531 |
| 81 | 6112 | 482 |
| 82 | 6036 | 434 |
| 83 | 5962 | 387 |
| 84 | 5888 | 341 |
| 85 | 5818 | 297 |
| 86 | 5751 | 255 |
| 87 | 5686 | 216 |
| 88 | 5629 | 181 |
| 89 | 5582 | 150 |
| 90 | 5545 | 125 |
| 91 | 5519 | 104 |
| 92 | 5504 | 86 |
| 93 | 5498 | 72 |
| 94 | 5500 | 59 |
| 95 | 5511 | 49 |

Supplemental figure 1c.

60%

|  | 5(60%) | 2(60%) |
| --- | --- | --- |
| 0 | 117980 | 117980 |
| 0 | 117980 | 117980 |
| 0 | 117980 | 117980 |
| 0 | 117980 | 117980 |
| 0 | 117980 | 117980 |
| 1 | 109596 | 108516 |
| 2 | 91605 | 85958 |
| 3 | 60955 | 67572 |
| 4 | 35700 | 26685 |
| 5 | 58171 | 28576 |
| 6 | 102675 | 99733 |
| 7 | 19289 | 29042 |
| 8 | 4268 | 1836 |
| 9 | 4176 | 1071 |
| 10 | 7849 | 1026 |
| 11 | 20173 | 1493 |
| 12 | 46980 | 2599 |
| 13 | 54641 | 5716 |
| 14 | 29563 | 15312 |
| 15 | 14663 | 29223 |
| 16 | 11910 | 23690 |
| 17 | 15843 | 15742 |
| 18 | 26730 | 13845 |
| 19 | 41320 | 14958 |
| 20 | 42599 | 15559 |
| 21 | 30011 | 14877 |
| 22 | 20817 | 14566 |
| 23 | 18711 | 15584 |
| 24 | 22115 | 17447 |
| 25 | 29755 | 18535 |
| 26 | 37018 | 17855 |
| 27 | 36638 | 16592 |
| 28 | 30188 | 16094 |
| 29 | 25066 | 16486 |
| 30 | 24099 | 17172 |
| 31 | 26938 | 17583 |
| 32 | 31941 | 17742 |
| 33 | 35544 | 17944 |
| 34 | 34731 | 18270 |
| 35 | 31132 | 18536 |
| 36 | 28396 | 18596 |
| 37 | 28259 | 18536 |
| 38 | 30521 | 18588 |
| 39 | 33608 | 18835 |
| 40 | 35250 | 19156 |
| 41 | 34399 | 19429 |
| 42 | 32320 | 19626 |
| 43 | 30920 | 19782 |
| 44 | 31088 | 19899 |
| 45 | 32622 | 19982 |
| 46 | 34441 | 20072 |
| 47 | 35198 | 20181 |
| 48 | 34478 | 20311 |
| 49 | 33135 | 20438 |
| 50 | 32368 | 20544 |
| 51 | 32704 | 20681 |
| 52 | 33824 | 20831 |
| 53 | 34897 | 20963 |
| 54 | 35170 | 21050 |
| 55 | 34603 | 21099 |
| 56 | 33831 | 21170 |
| 57 | 33521 | 21282 |
| 58 | 33887 | 21407 |
| 59 | 34635 | 21506 |
| 60 | 35229 | 21576 |
| 61 | 35287 | 21644 |
| 62 | 34880 | 21715 |
| 63 | 34421 | 21773 |
| 64 | 34291 | 21799 |
| 65 | 34575 | 21800 |
| 66 | 35042 | 21807 |
| 67 | 35348 | 21832 |
| 68 | 35294 | 21865 |
| 69 | 34984 | 21890 |
| 70 | 34704 | 21905 |
| 71 | 34676 | 21924 |
| 72 | 34907 | 21946 |
| 73 | 35215 | 21968 |
| 74 | 35378 | 21982 |
| 75 | 35310 | 21991 |
| 76 | 35106 | 22002 |
| 77 | 34956 | 22019 |
| 78 | 34981 | 22041 |
| 79 | 35160 | 22061 |
| 80 | 35359 | 22074 |
| 81 | 35441 | 22085 |
| 82 | 35372 | 22095 |
| 83 | 35232 | 22105 |
| 84 | 35145 | 22109 |
| 85 | 35182 | 22115 |
| 86 | 35309 | 22120 |
| 87 | 35427 | 22126 |
| 88 | 35457 | 22131 |
| 89 | 35391 | 22136 |
| 90 | 35293 | 22141 |
| 91 | 35245 | 22142 |
| 92 | 35277 | 22142 |
| 93 | 35361 | 22142 |
| 94 | 35428 | 22144 |
| 95 | 35431 | 22144 |
